# Adult ADHD Medications and Their Cardiovascular Implications

**DOI:** 10.1155/2016/2343691

**Published:** 2016-08-08

**Authors:** A. Sinha, O. Lewis, R. Kumar, S. L. H. Yeruva, B. H. Curry

**Affiliations:** ^1^Division of Cardiology, Saint Luke's University Health Network, 801 Ostrum Street, Bethlehem, PA 18015, USA; ^2^Division of Pulmonary Medicine, Department of Internal Medicine, Howard University Hospital, 2041 Georgia Avenue, Washington, DC 20060, USA; ^3^Department of Internal Medicine, Howard University Hospital, 2041 Georgia Avenue, Washington, DC 20060, USA; ^4^Division of Hematology & Oncology, Department of Internal Medicine, Howard University Hospital, 2041 Georgia Avenue, Washington, DC 20060, USA; ^5^Division of Cardiology, Howard University Hospital, 2041 Georgia Avenue, Washington, DC 20060, USA

## Abstract

Attention-deficit/hyperactivity disorder (ADHD) is a chronic neurobiological disorder exhibited by difficulty maintaining attention, as well as hyperactivity and impulsive behavior. Central nervous system (CNS) stimulants are the first line of treatment for ADHD. With the increase in number of adults on CNS stimulants, the question that arises is how well do we understand the long-term cardiovascular effects of these drugs. There has been increasing concern that adults with ADHD are at greater risk for developing adverse cardiovascular events such as sudden death, myocardial infarction, and stroke as compared to pediatric population. Cardiovascular response attributed to ADHD medication has mainly been observed in heart rate and blood pressure elevations, while less is known about the etiology of rare cardiovascular events like acute myocardial infarction (AMI), arrhythmia, and cardiomyopathy and its long-term sequelae. We present a unique case of AMI in an adult taking Adderall (mixed amphetamine salts) and briefly discuss the literature relevant to the cardiovascular safety of CNS stimulants for adult ADHD.

## 1. Introduction

Adult ADHD affects an estimated 3% to 5% of adults worldwide. It is seen in 4.4% (62% male, 38% female) of the US adult population [1]. CNS stimulants, namely, amphetamine based stimulants, methylphenidate, dextromethamphetamine, dextromethylphenidate, and modafinil, are the first line of treatment for ADHD. CNS stimulants act by inhibiting reuptake of norepinephrine and dopamine as well as increasing their release into the extracellular space. Approximately, 2% of American adults in 20–44 age groups were using stimulant medications for ADHD in 2010 which was a significant increase in use among both males and females (+188% and +265%, resp.) when compared to the prior decade [2]. In the study by Gerhard et al., 10.5% of adult ADHD patients had evidence of ≥1 preexisting cardiovascular condition. Even in patients with preexisting cardiovascular conditions, close to 80% of all ADHD medications initiated were stimulants. The knowledge of preexisting cardiovascular conditions did not significantly reduce the likelihood of stimulant treatment [3].

Adults with ADHD are possibly at higher risk for developing unhealthy lifestyle and cardiovascular risk factors, such as smoking and obesity, and have a greater probability of chronic substance misuse. Studies suggest that adult ADHD patients are more likely to experiment with licit and illicit substances, especially at earlier ages, and exhibit risky substance use patterns and subsequently develop substance use disorders [4, 5]. ADHD patients also have cooccurring psychiatric disorders, like anxiety (47%), mood disorder (38%), poor impulse control (20%), and substance use disorders (15%) [1]. These individuals can be on a number of medications along with stimulant drugs; effect of combination drug therapy on cardiovascular system has not been well studied.

It is possible that, in adults on CNS stimulant therapy, chances of having other cardiovascular risk factors may increase with each passing decade of life. Scrutiny has recently increased, primarily due to marked increase in the use of CNS stimulants among adults [6]. AMI as well as atrial fibrillation has been previously reported in cases of recreational amphetamine abuse [7] and also in adolescent ADHD patients on stimulant therapy [8]. Here, we discuss a rare case of AMI and atrial fibrillation in an adult who was on mixed amphetamine salts and briefly review pertinent literature.

## 2. Case

A 31-year-old Caucasian man presented to the emergency room with palpitations associated with shortness of breath and lightheadedness. He was diagnosed with ADHD at 15 years of age and was prescribed mixed amphetamine salts. His starting dose of mixed amphetamine salts was 7.5 mg and he took it for 5 years after the diagnosis. He restarted taking it 6 months prior to presentation as his symptoms were interfering with his daily activities. He had been prescribed 20 mg of mixed amphetamine salts twice daily but three months back had cut back on his dose to 20 mg once a day, on account of intermittent self-limiting episodes of palpitations lasting 5–10 minutes. He did not seek medical attention for these symptoms. On the day of presentation, he had taken mixed amphetamine salts 20 mg in the morning and later took 10 mg in the afternoon. However, that evening he also consumed four 16-ounce beers but denied any smoking or use of illicit drugs. After about 1-2 hours, he developed significant palpitations that were different in nature and lasted longer along with shortness of breath and lightheadedness without any chest pain, nausea, or diaphoresis.

**Figure 1:**
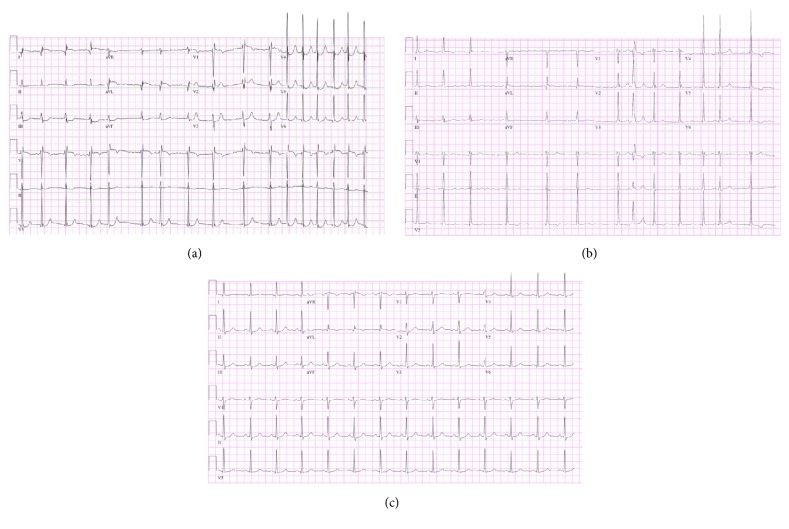
Patient's EKGs showing (a) atrial fibrillation with ST elevations in leads I, aVL, V1, and V2 with reciprocal changes in leads III and aVF, (b) atrial fibrillation with resolution of ST segment elevations, and (c) normal sinus rhythm. Normal EKG.

His past medical history was unremarkable except for ADHD. He did not have other symptoms or signs suggestive of pheochromocytoma or hyperthyroidism. There was no family history of cardiovascular diseases. He admitted occasional use of alcohol and marijuana (once a month) but denied the use of energy drinks or other illicit drugs. He had no known drug allergies and took no other medications other than mixed amphetamine salts. On physical examination, his heart rate was 92 beats per minute, respirations were 20, and blood pressure was 90/53 mm of Hg. His cardiac exam was significant for irregularly irregular heart sounds of varying intensity. His lung fields were clear and he was alert and oriented to person, place, and time. A 12-lead electrocardiogram (EKG) revealed atrial fibrillation with an average ventricular response of 98 bpm and ST elevations in leads I, aVL, V1, and V2 with reciprocal changes in leads III and aVF (Figure 1(a)). The EKG after 13 minutes showed resolution of the ST segment elevations (Figure 1(b)).

His initial set of cardiac enzymes was CPK: 138 U/L, CK-MB: 5.9 ng/mL, troponin I: 0.13 ng/mL, and myoglobin: 4.7 ng/mL. He received aspirin 325 mg. He was later admitted to the cardiac unit for hemodynamic monitoring and serial cardiac enzymes, EKGs, and assessment of left ventricular (LV) function. Subsequent troponin levels rapidly increased to 6.91 ng/mL. The rest of his cardiac enzymes were CPK: 387 U/L, CK-MB: 77.9 ng/mL, and myoglobin: 34 ng/mL. Chest X-ray was normal. All of his routine laboratories including lipid profile were unremarkable. His TSH was 1.83 U/mL and free T4 was 0.91 ng/dL. The urine drug screen was positive for amphetamines only. A transthoracic echocardiogram showed normal LV dimensions and good LV systolic function, without evidence of wall motion abnormalities. He also underwent coronary angiography that did not reveal any epicardial coronary artery disease. He was treated with oral diltiazem 30 mg three times daily and the patient's symptoms and atrial fibrillation resolved (Figure 1(c)). Serum troponin levels started trending down by second day. The final diagnosis of AMI and atrial fibrillation secondary to mixed amphetamine salts use was made. Patient had uneventful hospital course and was discharged on third day.

## 3. Discussion

Adderall, formulation of D-amphetamine and L-amphetamine salts in the ratio of 3 : 1, is a prescription CNS stimulant drug for ADHD and is used in all age groups. The exact mechanism of cardiovascular impact of stimulants is unknown. The proposed mechanisms by which stimulants may give rise to adverse cardiovascular events are (a) by elevating blood pressure (BP) and increasing the heart rate (HR) [9], (b) increased levels of circulating catecholamines inducing vasospasm [10], (c) higher levels of circulating proinflammatory immunoactive glycation end products causing vasculitis [11], (d) inducing QT interval prolongation which is associated with torsades de pointes [12], and (e) coronary artery intimal hyperplasia [13].

Amphetamines have acute chronotropic and pressor effects. The cardiovascular epidemiological literature has shown that even modest increases in BP and HR have been associated with increased risk of adverse cardiovascular events [14–17]. In the meta-analysis carried out by Mick et al. on 2665 adult patients, it was observed that CNS stimulants used for adult ADHD were associated with a statistically significant increase in resting heart rate of 5.7 bpm and increase in systolic blood pressure of 1.2 mmHg but not of diastolic blood pressure. A low overall risk (≤5%) of clinically significant cardiovascular events, including tachycardia or hypertension, was also observed [18]. Epidemiological studies have demonstrated that elevated resting heart rate is a significant independent predictor of mortality and a shorter life expectancy [16, 17]. Cooney et al. demonstrated that 15 bpm increase in heart rate was found to increase the rate of cardiovascular disease mortality by 23–50% in men and women [16]. Perret-Guillaume et al. showed that heart rate increase of 10 bpm is associated with a 20% increased risk of cardiac death [17]. HR increases comparable with those observed with CNS stimulant treatment for adult ADHD have been associated with a 17% increased cardiovascular mortality [19] and about 8% in those with coronary artery disease [20]. In another study carried out by Wilens et al., statistically significant change in systolic blood pressure by about 5 mm Hg and diastolic blood pressure of about 7 mm Hg was observed. Blood pressure variations of this magnitude, in particular during long-term therapy, have been acknowledged to increase morbidity and mortality [21].

Abnormal sympathetic and parasympathetic cardiac inputs secondary to stimulant use can result in increased myocardial excitability and conductance. Patients with exaggerated sympathetic nervous system activity are more susceptible to develop clinically significant cardiac arrhythmias typically more in the setting of an underlying structural heart defect [22]. There are very few reports associating amphetamine-dextroamphetamine therapy to new onset atrial fibrillation [23] and atrial flutter [24]. A recent case series analysis study conducted in 1224 patients aged <17 years showed increased risk of arrhythmia in all exposed time periods (incidence rate ratio 1.61, 95% confidence interval 1.48 to 1.74), and the risk was highest in the children with congenital heart disease. The risk of myocardial infarction was higher between 8 and 56 days after the start of treatment with methylphenidate. Overall, for all exposed time periods there was no significant risk of myocardial infarction (1.33, 0.90 to 1.98). There was no significant increased risk for hypertension, ischemic stroke, or heart failure [25].

Other CNS stimulants such as modafinil and methylphenidate have also been rarely reported to cause frequent premature ventricular contractions [26]. Zhang et al. showed that long QT syndrome patients especially males, when treated with ADHD medications, had higher risk of unfavorable cardiac events during follow-up. The stimulant drugs did not significantly change average QTc interval. Small proportion of individuals in the study cohort however did have increment in QTc by 30–60 milliseconds to above 470–500 milliseconds [27].

The three most important observational studies conducted on adults that address whether prescription stimulant and nonstimulant drugs are associated with adverse cardiovascular events are by Holick et al., Schelleman et al., and Habel et al. [28–30]. Holick et al. conducted their study on matched cohort of 21,606 stimulant ADHD medication initiators, 21,606 atomoxetine initiators, and 42,993 age and sex matched general population cohort (greater than or equal to 18 years of age) between 2003 and 2006. They observed an increased risk of transient ischemic attack only and not of stroke, among adult ADHD medication starters when compared to the general population, in their secondary analysis. However, unlike the primary analysis the general population cohort was not matched with the combined atomoxetine and prescription stimulant user cohort using propensity matching [28].

Schelleman et al. matched 43,999 new methylphenidate users to 175,955 nonusers in adults (greater than or equal to 18 years of age) and reported that methylphenidate is associated with a 1.8- fold increased risk of sudden death or ventricular arrhythmia, but no similar trend was noted for stroke, myocardial infarction, or combined stroke/myocardial infarction. The drawback with this study was that primary analyses were adjusted for age and data source only. There was smaller but significantly increased risks of sudden death, ventricular arrhythmia, and all-cause death, similar to primary analyses, with subsequent analyses using propensity scores to address confounding factors [29].

Most extensive and largest of the three adult studies carried out by Habel et al. did not report any increased risk of myocardial infarction, sudden cardiac death, and stroke among 150,359 adults ADHD prescription stimulant users during short median exposure (0.33 years) when compared to 292,839 nonusers in adults aged 25 years to 64 years. Surprisingly, statistical results suggested that ADHD medications have protective effect with regard to serious cardiovascular outcome which according to the authors themselves is biologically implausible and most likely could be attributed to be biased by a “healthy user effect” [30]. Patient pool in the above three observational studies was less selective as compared to randomized clinical trial but certainly healthier than the general adult ADHD population. The biggest limitation of Habel et al.'s study is consideration of only severe cardiovascular events that dampened the absolute conclusion in spite of big sample size.

These results were similar to two other studies that were conducted in children and young adults. McCarthy et al. showed no increase in risk of sudden death associated with stimulants and atomoxetine in patients aged 2–21 years [31]. Cooper et al. conducted a retrospective cohort study in children and young adults aged 2–24 years and showed that current users of ADHD drugs were not at increased risk for serious cardiovascular events, although the upper limit of the 95% confidence interval indicated that a doubling of the risk could not be ruled out. However, the absolute magnitude of such an increased risk would be low [32].

Safety concerns with regard to prescriptions stimulants use have guided governmental regulatory policy from time to time. Current clinical recommendations emphasize the need to assess the patient's personal and family cardiac history prior to initiation of ADHD pharmacotherapy, being vigilant about abnormal cardiovascular history (e.g., premature sudden/unexpected death in children or young adults, clinically important arrhythmias, prolonged QT syndrome, hypertrophic cardiomyopathy, and Marfan syndrome) [33]. HR and BP should be measured before initiating medications and routinely during treatment. Universal EKG screening has not been shown to be cost effective, as it has not demonstrated any incremental benefit in preventing sudden cardiac death in children with ADHD [34]. EKG screening and cardiologist consultation are recommended for ADHD patients who have positive history of structural cardiac abnormalities [35] and even though baseline EKG before initiating ADHD medications is considered reasonable by ACC/AHA [36], it is not endorsed by American Academy of pediatrics [33].

Ours is the first case report of AMI and atrial fibrillation in an adult who was on short acting mixed amphetamine salts without intention to overdose or abuse, to the best of our knowledge. Patient reduced his earlier dose of 20 mg of mixed amphetamine salts twice daily to 20 mg once daily. He did however consume higher than his daily total dose (30 mg ← 20 mg) of mixed amphetamine salts on the day of presentation. The temporal association of AMI and atrial fibrillation with higher than usual dose of mixed amphetamine salts makes amphetamines the most plausible etiology. It is difficult to ascertain whether atrial fibrillation occurred independently or it was exacerbated by higher than usual dose of stimulant. The simultaneous use of alcohol with stimulants may have had additive effects. Cytochrome P450 enzymes in the liver areinvolved in metabolism of both alcohol and amphetamine. Alcohol competes with amphetamine for the metabolism in the liver and hence may enhance the bioavailability of amphetamine [37, 38]

In an adult stimulant user, in absence of cardiovascular risk factors, coronary vasospasm either epicardial or microvascular may be a predominant reason for AMI. Such patients usually present with atypical chest pain. The diagnosis of AMI involves elevation of myocardial necrosis biomarkers, ischemic symptoms, and EKG changes. Management of such patients is guided by clinical presentation and initial imaging studies employed to assess left ventricular function and coronary patency. Those with persistent chest pain, hemodynamic instability, and rising troponin levels should be managed on a case-by-case basis.

Adult ADHD is significantly impairing and the use of CNS stimulants among adults is on the rise. It is likely that in coming years increasing number of unhealthy adult ADHD population is going to be exposed to CNS stimulant treatment. Definite conclusions regarding the overall cardiovascular safety of these agents cannot currently be made for patients at varying risk for cardiovascular disease or in relation to chronic treatment exposure. At present, there is less data available to guide and inform clinical practice in many patients with adult ADHD and concomitant cardiovascular disease and/or risk factors.

## 4. Conclusion 

The ongoing debate about the cardiovascular safety of these medications is here to stay and underscores the challenge in conclusively establishing safety and the need to evaluate risk in the context of the evolving clinical practice. Prospective scientific investigations should include systemic cardiovascular monitoring in clinical trials, more extensive and prolonged cardiovascular studies, and large-scale epidemiologic studies to help gain insight with regard to both the short- and longer-term cardiovascular impact of CNS stimulants in adults with ADHD.
